# Optical bench evaluation of the effect of pupil size in new generation monofocal intraocular lenses

**DOI:** 10.1186/s12886-023-02839-y

**Published:** 2023-03-20

**Authors:** Aixa Alarcon, Carmen Canovas, Bram Koopman, Milind V Pande, Douglas D Koch, Patricia Piers

**Affiliations:** 1 Johnson and Johnson Vision, Groningen, Netherlands; 2 Vision Surgery & Research Centre, North Ferriby, East Yorkshire, UK; 3 Cullen Eye Institute, Baylor College of Medicine, Houston, US

**Keywords:** Cataract, Intraocular lens, Monofocal, Intermediate, Enhanced monofocal

## Abstract

**Background:**

A new generation of enhanced monofocal IOLs has been introduced to slightly increase the depth of focus as compared to standard monofocal IOLs. The purpose of this study is to evaluate the effect of pupil size on the through-focus optical performance of three new enhanced monofocal IOLs, designed to improve the range of vision as compared to standard monofocal IOLs.

**Methods:**

Optical bench testing in white light was performed for different pupils, using an average cornea eye. Distance image quality was evaluated using Modulation Transfer Function (MTF) measurements. Through-focus Visual Acuity (VA) was simulated from these measurements (sVA). Three enhanced monofocal IOLs (ICB00, ISOPure, and RayOne-EMV) and three standard monofocal IOLs: two aspheric (ZCB00 and SN60WF) and one spherical (AAB00) were included.

**Results:**

The enhanced monofocal IOLs provided an improvement in the intermediate sVA as compared to standard monofocal IOLs. For ICB00, the improvement was independent of the pupil size, while for the ISOPure and RayOne-EMV, the intermediate sVA improved with increased pupil size. Similar to the spherical monofocal IOL, the ISOPure and RayOne-EMV showed a strong correlation between improvement in intermediate sVA and reduction of distance sVA and MTF, and increasing pupil size. ICB00 provided the same distance sVA as the aspheric monofocal IOLs and the lowest variability in MTF with pupil size.

**Conclusion:**

Optical bench results showed that the ISOPure and RayOne-EMV provide similar performance to a spherical monofocal IOL, with a strong pupil dependency for distance and intermediate vision. The other enhanced monofocal IOL, ICB00, provided a sustained improvement in simulated intermediate VA and maintained distance image quality comparable to that of the standard aspheric monofocal IOLs, even for larger pupils.

## What was known

- Distance MTF is reduced with larger pupil size in eyes with imperfect correction of SA.
- New generation enhanced monofocal IOLs can increase the depth of focus.

## What this paper adds

- The effect of the pupil size on the optical image quality and simulated visual acuity of different monofocal IOLs depends on the optical technology and the correction of corneal spherical aberration.
- The new generation monofocal IOL, designed to improve the depth of focus while fully correcting corneal spherical aberration, shows a pupil-independent improvement in simulated intermediate visual acuity and maintained distance visual acuity compared to conventional monofocal IOLs.

## Introduction

The standard of care in cataract surgery is monofocal intraocular lenses (IOLs). The current monofocal lenses comprise a variety of different optical concepts, which can be characterized by the optical design, that determines for example the amount of Spherical Aberration (SA) that is being induced by the optic, the spectral filtering properties, the chromatic dispersion of the material, and, of course, the geometrical design (e.g., sharp optic edge, haptic design, and angulation). The amount of SA is of particular interest, as it interacts with the SA of the patient’s cornea and thus determines the achievable optical resolution and depth of focus. There are mainly three different IOL concepts on the market based on SA. The first group is conventional spherical IOLs, which feature a spherical optical surface and therefore induce a positive amount of SA by design. The second group of IOLs uses aspheric optical surfaces with zero asphericity to eliminate this intrinsic SA of the IOL. These IOLs are referred to as “aberration neutral IOLs”. The third group of IOLs employ aspheric surfaces as well, but are designed to induce a negative amount of SA in order to (partially or fully) compensate for the cornea’s SA, referred to as “aberration correcting IOLs”. This third group of lenses was described by Holladay in 2002 [1] and has been demonstrated to reduce ocular SA to near-zero and significantly improve the contrast sensitivity at distance compared to standard spherical monofocal IOLs[2–5].

Recently, a new generation of enhanced monofocal IOLs has been introduced to slightly increase the depth of focus as compared to standard monofocal IOLs; these include the TECNIS Eyhance, model ICB00 (Johnson & Johnson Vision, Santa Ana, USA)[6], the RayOne EMV (Rayner Intraocular Lenses Limited, Worthing, UK), and ISOPure 1.2.3 (PhysIOL S.A, Liege, Belgium)[7]. In addition, currently, there are numerous IOL designs available in the market that use refractive technologies to extend the depth of focus. These refractive technologies are designed to improve uncorrected reading vision without the typical side effects (e.g. halo, starbursts, and glare) of diffractive concepts. However, although refractive designs might bring some advantages to reduce photic phenomena, they are also associated with more pupil dependency, and distance image quality reduction, than diffractive designs.

In this study, we used optical bench testing to evaluate the effect of pupil size on through-focus optical performance and distance image quality of three new-generation enhanced monofocal IOLs. The results were compared to those of standard monofocal IOLs with different levels of SA and chromatic aberration.

## Methods

A total of six IOL models, with different optical designs and materials, were evaluated as listed in Table 1. The TECNIS Eyhance, model ICB00, and the ISOPure 1.2.3 use non-diffractive technologies and are defined by polynomial-based surfaces that are designed to increase depth of focus with minimum compromises at distance. The RayOne EMV is an aspheric monofocal IOL with positive spherical aberration designed to provide improved intermediate vision when implanted with 1.0D offset (monovision). These three IOL models were selected to provide a wide representation of the new category of enhanced monofocal IOLs, with different optical designs and materials. Additionally, to illustrate the expected performance of the standard monofocal IOLs, two aspheric and one spherical IOL were also included in the analysis: two aspheric (TECNIS Monofocal, model ZCB00, and the Acrysof IQ Monofocal, model SN60WF) designed to fully or partially compensate corneal spherical aberration and one spherical IOL (the Sensar 1-pc, model AAB00).

Modulation transfer function (MTF) and the phase transfer function (PTF) were measured in water at room temperature with white light and for a wide range of pupil diameters (2, 3, 4, and 5 mm). The IOLs were measured in a custom made physical model eye [8] that follows the a ISO 11979-2:2014 model eye 2. This eye model was selected because it resembles the spherical aberration of the average cornea (0.28 micrometers for a 6 mm entrance pupil as reported by Wang et al. [9]) as well as the average chromatic aberration of the human cornea (~ 1D for chromatic aberration between 450 and 650 nm). In order to derive a clinically meaningful metric for the image quality, the MTF and PTF measurements were used to calculate the simulated binocular visual acuity (sVA) using weighted Optical Transfer Function (wOTF) metric and the methodology described by Alarcon et al. [10]. Because the coefficients to convert wOTF to simulated VA were only provided for a 3 mm pupil only, sVA for other pupil sizes (2, 4 and 5 mm) was provided relative to the standard aspheric monofocal IOL, model ZCB00. This was required to balance the impact of the coefficients for the different pupil sizes.

To simulate the effect of different lighting conditions, the best focus position (0D) was found for an average photopic pupil of 3 mm was maintained during the measurement at the other different pupil sizes.

**Table 1:** Intraocular lens models under evaluation

Manufacturer	Brand name (model)	IOL Spherical Aberration	Abbe number	Longitudinal chromatic aberration (450-650 nm)
Johnson & Johnson Surgical Vision; Santa Ana CA, USA	TECNIS Eyhance (ICB00)	-0.27 μm;^10^	55	1.30 D
	TECNIS Monofocal (ZCB00)	− 0.27 μm ^1^	55	1.30 D
	Sensar 1-piece (AAB00)	Positive SA, power dependent	55	1.30D
Alcon Laboratories, Forth Worth TX, USA	AcrySof® IQ Monofocal (SN60WF)	-0.20 μm	< 37 [11]	1.77D
PhysIOL, Liege, Belgium	ISOPure 1.2.3 (ISOPure)	-0.11 μm	42 [12]	not reported
Rayner Intraocular Lenses Limited, Worthing, UK	RayOne-EMV	Positive SA	56 [12]	not reported

## Results

### Simulated visual acuity

**Fig. 1:**
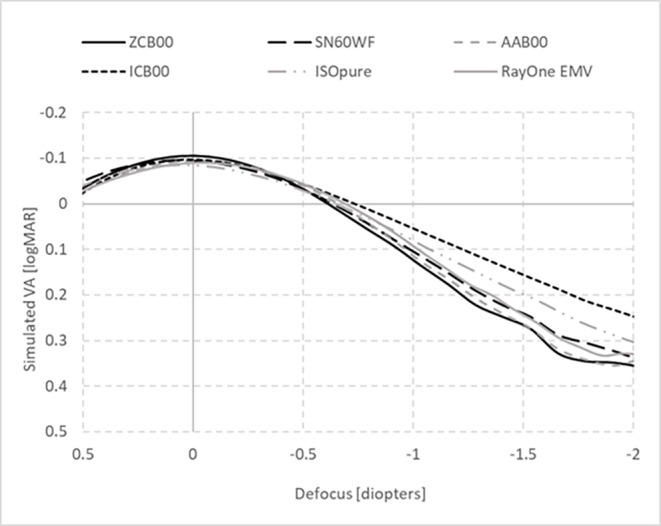
Simulated binocular VA defocus curves of different monofocal IOLs for 3 mm pupil

Figure 1 shows the defocus curves based on simulated binocular VA for the 6 different lenses for an average photopic pupil of 3 mm. As Fig. 1 illustrates, RayOne EMV provides a range of vision comparable to that of a standard monofocal IOL (AAB00, ZCB00 or SN60WF), while ICB00 and ISOpure provide an improved range of vision, especially around − 1.5D.

**Fig. 2:**
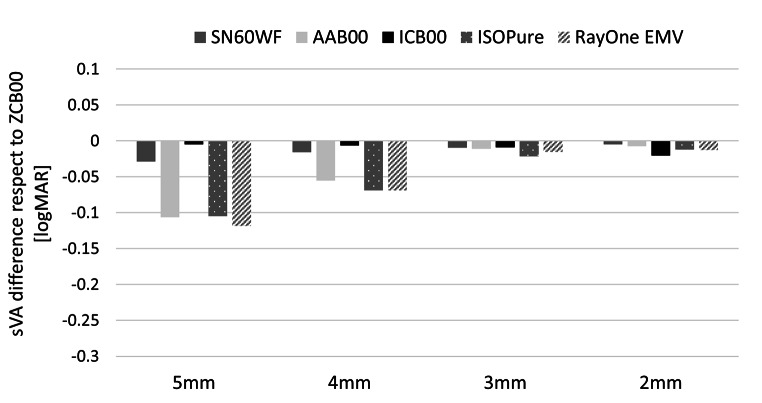
Simulated VA at far (0D) with respect to the aspheric monofocal IOL ZCB00 from 5 to 2 mm pupil. Negative values indicate worse VA than the aspheric monofocal IOL.

**Fig. 3:**
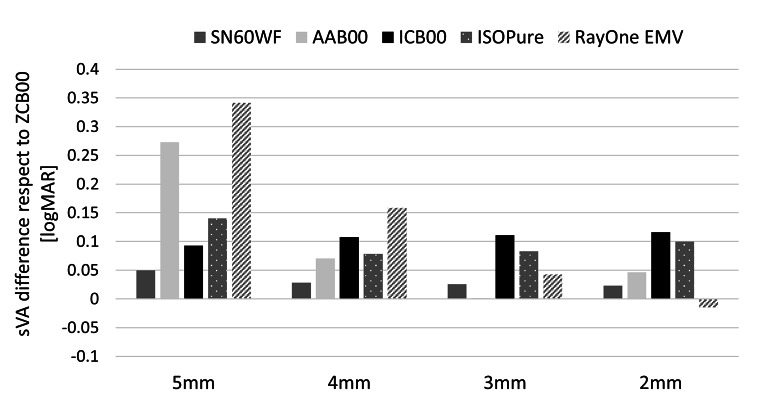
Simulated VA at intermediate (-1.5D) with respect to the aspheric monofocal IOL ZCB00 from 5 to 2 mm pupil. Positive values indicate better VA than the aspheric monofocal IOL.

The effect of pupil size on sVA at far (0 D) and intermediate (-1.5 D) defocus points, relative to the standard aspheric monofocal IOL ZCB00, is shown in Figs. 2 and 3 respectively. As expected, the standard aspheric monofocal SN60WF, which partially compensates for corneal spherical aberration, has similar sVA performance as ZCB00 with an average difference below 0.05 logMAR for intermediate and distance sVA. The standard spherical monofocal AAB00 and the new generation enhanced monofocals, ISOPure and RayOne EMV, showed a similar correlation between a drop in far sVA and improvement in intermediate sVA for larger pupils. The enhanced monofocal ICB00 provided the lowest pupil variation, with no difference with respect to ZCB00 at far, and 0.1 logMAR improvement at intermediate for all pupil sizes.

### Distance image quality

**Fig. 4:**
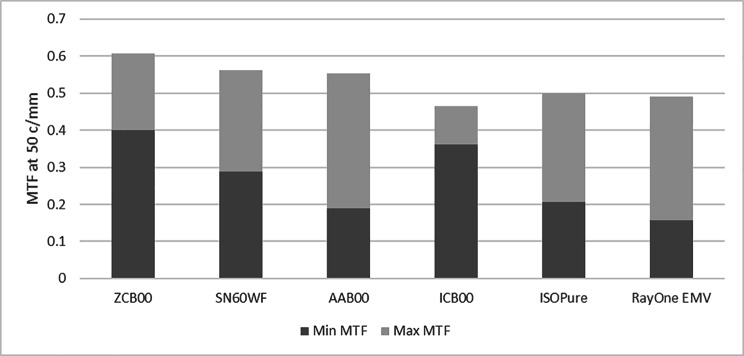
Minimum (Min) and maximum (Max) MTF at 50 c/mm at far in the pupil range between 2 and 5 mm. Minimum MTF for all IOLs was for 5 mm pupil. Maximum MTF was found for different pupil sizes depending on the IOL.

Figure 4 provides the ranges of the MTF at 50 c/mm between 2 and 5 mm pupils for the 6 different IOL models. The difference between the minimum and the maximum MTF provides an estimation of the variability in distance image contrast with the pupil size. This variation between the maximum and minimum MTF values of the three standard monofocal IOLs, ZCB00, SN60WF and AAB00, was directly related to the level of corneal spherical aberration correction provided by each of the designs (see Table 1). The spherical monofocal IOL, model AAB00, and the new generation enhanced IOLs (ISOpure and RayOne EMV) provided the highest pupil dependency in distance image quality, with a relative drop of more than 50% from the maximum to the minimum MTF level. The aspheric monofocal IOL, model ZCB00, and the new generation enhanced monofocal ICB00 provided the lowest variability with pupil size and highest minimum MTF values.

**Fig. 5:**
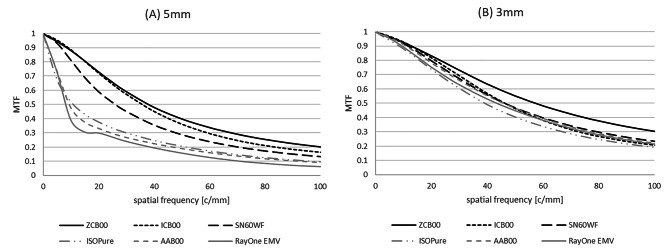
Through frequency MTF curves at far (0D) for 5 mm (A) and 3 mm (B) pupil

As illustrated in Fig. 5A, models AAB00, ISOPure and RayOne EMV also provided the lowest MTF levels for a standard mesopic pupil (5 mm), with large losses in image contrast specially for lower spatial frequencies. For a standard photopic pupil of 3 mm (Fig. 5B), all IOLs provided similar MTF values except for model ZCB00, which has the highest MTF levels at all spatial frequencies.

## Discussion

Optical bench results provide a good estimation of the clinical performance of intraocular lenses when performed in clinically relevant conditions (cornea that resembles the spherical and chromatic aberration of the real cornea and white light) and using clinically relevant pre-clinical metrics[10, 13–15]. The additional value of optical bench studies, compared to clinical investigations, is that they provide a direct comparison under the exact same conditions between different IOL models without the intrinsic variability associated with clinical studies. In this study, the effect of the pupil size was evaluated for 3 standard monofocal IOLs (2 aspheric aberration correcting models and one spherical model) and 3 new generation monofocal IOLs with different levels of spherical aberration and different chromatic performance.

While SA is the most important monochromatic aberration in the human eye, chromatic aberrations also play an important role for optical performance, especially in the pseudophakic eye, and there are significant differences in the chromatic dispersion of current materials used for IOLs[11, 16]. To account for this, measurements were performed in white light, using an eye model with the same average spherical and chromatic aberration of the average cornea.

The results of this optical bench study show that the new generation monofocal IOL, model ICB00, provided an extended range of focus and an improvement in simulated intermediate VA relative to standard aspheric monofocal IOL, while maintaining the same distance sVA. This is in agreement with the outcomes of the meta-analysis study of Wan et al. [17] summarizing the results of 12 different clinical studies comparing ICB00 to standard monofocal IOLs that found that ICB00 provides an improvement in unaided intermediate vision with similar distance performance. Other clinical studies have shown a significant improvement in intermediate VA in patients implanted with the ICB00 IOL compared to the aspheric monofocal lens ZCB00 but no significant differences at distance [18–25]. Cinar et al. compared clinical results of the SN60WF with the ICB00 and found superior VA for intermediate vision for the ICB00 and no significant differences at distance and near VA [26]. This consistent clinical performance of the ICB00 can be explained by the pupil independent design of this IOL model.

Stodulka et al. [7] reported clinical results on the ISOPure IOL. Although no control lens was used on this study, the mean binocular DCIVA of the ISOPure IOL was 0.2 logMAR, which is between 0.1 and 0.05 logMAR lower than the DCIVA reported for the ICB00 in different studies [18–20] and in good agreement with pre-clinical simulations. In contrast, the distance VA was slightly better for the ISOPure (-0.09 ± 0.06 logMAR) compared to the results reported for the ICB00 (ranging between − 0.06 ± 0.01 logMAR [19] and 0.03 ± 0.09 logMAR [23]).

Models ICB00 and ZCB00 provided consistently high MTF levels as expected, as both models fully compensate for the SA of the average cornea, and the effect of correcting spherical aberration is especially evident with large pupils [6]. Vega et al. [27] demonstrated that the ICB00 and ZCB00 showed comparable MTF levels and pupil independent optical performance, which supports our findings. This is in alignment with the clinical results of Mencucci et al. that did not find significant differences between patients implanted with ICB00 and ZCB00 when using objective image quality metrics like the Strehl ratio and the MTF cut-off frequency [18]. Additionally, Mencucci et al. [18] and Auffarth et al. [19] also measured the same contrast sensitivity in ICB00 and the ZCB00 patients.

The other new generation monofocal IOLs, ISOPure and RayOne EMV, provided MTF values at 5 mm comparable to the spherical monofocal IOL AAB00. Labuz et al. [12] compared four different refractive EDOF IOLs to the ZCB00 in an optical-bench study, including ICB00, ISOPure, RayOne-EMV, and the AE2UV/ZOE (an aspheric IOL with increased aberrations in the central part of the lens). They also found lower MTF values at 4.5 mm for the ISOPure and RayOne EMV than ICB00. The image performance for distance vision and the halo/glare pattern of the ICB00 and the AE2UV/ZOE was found to be comparable to that of the ZCB00. The ICB00 showed best overall area under the simulated defocus curve, followed by the AE2UV/ZOE and the ISOPure. The halo/glare pattern for the ISOPure and the RayOne EMV were found to be significantly larger than for the ZCB00. Although halo and scatter were not evaluated in our study, previous optical bench studies have also shown no differences between ICB00 and ZCB00 [6, 27].

The effect of pupil size on distance image quality is affected by the metric used and the light conditions. Monochromatic light should not be used to compare the performance of different intraocular lenses made of different materials, as it underestimates the effect of chromatic aberration on the image quality [28]. Additionally, although a single spatial frequency of MTF can provide a relative estimation of the contrast sensitivity expected in the patients, it does not correlate with perceived visual acuity and therefore is not a good metric to predict through-focus performance. There are numerous integral metrics that were developed to either correlate with visual acuity, contrast sensitivity, depth of focus or other visual performance scores [29–32]. Some of them incorporate the estimated neural transfer function, and others can be differentiated between monochromatic and polychromatic metrics. Additionally, the coefficients to convert the metrics to visual acuity were found for an average pupil size of 3 mm using different intraocular lens designs (monofocal, multifocal and extended depth of focus IOLs) made in different materials. Therefore, to evaluate other pupil sizes, these coefficients may not provide a good estimation of the expected visual acuity. For that reason, in our study absolute values of simulated visual acuity were only provided for 3 mm pupil, and for other pupil sizes a relative comparison was performed. The choice of the proper metric and the pupil dependency of the coefficients are therefore important and must be mentioned as a potential limitation of the current study. Another limitation of this study is that measurements were collected for an eye model with the average corneal spherical aberration. However, corneal spherical aberration is different for every eye and the outcomes of the IOL will change with corneal spherical aberration of the patient. Future studies could investigate the impact of different levels of corneal spherical aberration (or other aberrations). In addition, future studies should also consider the impact that other factors, like tilt and decentration of the IOL, can have in the performance of these designs.

## Conclusion

New generation monofocal IOLs provide an improvement in depth of focus as compared to standard monofocal IOLs. The effect of pupil size on through focus performance and distance image quality depends on the IOL design and the level of correction of corneal SA. Distance MTF was mainly reduced for larger pupil sizes in monofocal IOLs that do not fully compensate for corneal spherical aberration, such as the new generation monofocal IOL ISOPure and RayOne EMV and the spherical monofocal IOL AAB00. On the other hand, designs that fully compensate for corneal SA such as model ICB00 and ZCB00, showed the lowest variability with the pupil for both distance (simulated visual acuity and MTF) and intermediate sVA.

## Data Availability

All the information and figures available for publication is contained in this manuscript. Raw data won’t be disclosured. For more data/information related to the data, contact the corresponding author: Aixa Alarcon (aalarco4@its.jnj.com).
